# Cerebral Perfusion in Hemodialysis Patients: A Feasibility Study

**DOI:** 10.1177/20543581211010654

**Published:** 2021-05-06

**Authors:** Jessica Anne Vanderlinden, Rachel Mary Holden, Stephen Harold Scott, John Gordon Boyd

**Affiliations:** 1 Centre for Neuroscience Studies, Queen’s University, Kingston, ON, Canada; 2 Division of Nephrology, Department of Medicine, Queen’s University, Kingston, ON, Canada; 3 Department of Biomedical and Molecular Sciences, Queen’s University, Kingston, ON, Canada; 4 Division of Neurology, Department of Medicine, Queen’s University, Kingston, ON, Canada; 5 Department of Critical Care Medicine, Queen’s University, Kingston, ON, Canada

**Keywords:** end-stage kidney disease, the Repeatable Battery for the Assessment of Neuropsychological Status, Kinarm, neurocognitive impairment, hemodialysis, cerebral oximetry

## Abstract

**Background:**

Patients on hemodialysis (HD) are known to exhibit low values of regional cerebral oxygenation (rSO2) and impaired cognitive functioning. The etiology of both is currently unknown.

**Objective:**

To determine the feasibility of serially monitoring rSO2 in patients initiating HD. In addition, we sought to investigate how rSO2 is related to hemodynamic and dialysis parameters.

**Design:**

Prospective observational study.

**Setting:**

Single-center tertiary academic teaching hospital in Ontario, Canada.

**Participants:**

Six patients initiating HD were enrolled in the study.

**Methods:**

Feasibility was defined as successful study enrollment (>1 patient/month), successful consent rate (>70%), high data capture rates (>90%), and assessment tolerability. Regional cerebral oxygenation monitoring was performed 1 time/wk for the first year of dialysis. A neuropsychological battery was performed 3 times during the study: before dialysis initiation, 3 months, and 1 year after dialysis initiation. The neuropsychological battery included a traditional screening tool: the Repeatable Battery for the Assessment of Neuropsychological Status, and a robot-based assessment: Kinarm.

**Results:**

Our overall consent rate was 33%, and our enrollment rate was 0.4 patients/mo. In total 243 rSO2 sessions were recorded, with a data capture rate of 91.4% (222/243) across the 6 patients. Throughout the study, no adverse interactions were reported. Correlations between rSO2 with hemodynamic and dialysis parameters showed individual patient variability. However, at the individual level, all patients demonstrated positive correlations between mean arterial pressure and rSO2. Patients who had more than 3 liters of fluid showed significant negative correlations with rSO2. Less cognitive impairment was detected after initiating dialysis.

**Limitation:**

This small cohort limits conclusions that can be made between rSO2 and hemodynamic and dialysis parameters.

**Conclusions:**

Prospectively monitoring rSO2 in patients was unfeasible in a single dialysis unit, due to low consent and enrollment rates. However, rSO2 monitoring may provide unique insights into the effects of HD on cerebral oxygenation that should be further investigated.

**Trial Registration:**

Due to the feasibility nature of this study, no trial registration was performed.

## Abrégé

**Contexte:**

Les patients sous hémodialyse (HD) sont connus pour présenter de faibles valeurs de saturation cérébrale régionale (rSO2) et des fonctions cognitives altérées. L’étiologie de ces deux affections est actuellement inconnue.

**Objectifs:**

Vérifier la faisabilité d’un suivi en série de la rSO2 chez des patients ayant amorcé des traitements d’HD. Nous souhaitions également étudier la façon dont la rSO2 est liée aux paramètres hémodynamiques et de dialyse.

**Type d’étude:**

Étude observationnelle prospective.

**Cadre:**

Un centre hospitalier universitaire de soins tertiaires situé en Ontario, au Canada.

**Sujets:**

L’étude porte sur six patients ayant amorcé des traitements d’HD.

**Méthodologie:**

La faisabilité a été définie par un recrutement efficace à l’étude (plus d’un patient/mois), un taux de consentement élevé (au-delà de 70 %), des taux de saisie de données élevés (au-delà de 90 %) et la tolérance de l’évaluation. La rSO2 a été mesurée une fois par semaine pendant la première année de dialyse. Une batterie neuropsychologique a été réalisée trois fois au cours de l’étude: avant l’initiation de la dialyse, puis trois mois et un an après l’initiation de la dialyse. La batterie de tests neuropsychologiques comprenait un outil de dépistage traditionnel: le *Repeatable Battery for the Assessment of Neuropsychological Status*, et une évaluation robotisée: Kinarm.

**Résultats:**

Le pourcentage global de consentement était de 33 % et le taux de recrutement était de 0,4 patient/mois. En tout, 243 mesures de la rSO2 ont été enregistrées, avec un taux de saisie des données de 91,4 % (222/243), chez les 6 patients inclus à l’étude. Aucune interaction indésirable n’a été signalée en cours d’étude. Les corrélations entre la rSO2 et les paramètres hémodynamiques et de dialyse étaient variables d’un patient à l’autre. Tous les patients ont cependant montré une corrélation positive entre la pression artérielle moyenne et la rSO2 d’un point de vue individuel. Des corrélations négatives significatives avec la rSO2 ont été observées chez les patients ayant plus de trois litres de liquides. Moins de troubles cognitifs ont été détectés après l’amorce de la dialyse.

**Limites:**

La faible taille de la cohorte limite les conclusions pouvant être tirées sur les liens entre la rSO2 et les paramètres hémodynamiques et de dialyse.

**Conclusion:**

Le suivi prospectif de la rSO2 chez les patients s’est avéré irréalisable dans cette unité de dialyse en raison des faibles taux de consentement et de recrutement. Le suivi de la rSO2 pourrait cependant fournir une perspective unique sur les effets de l’HD sur la saturation cérébrale, laquelle devrait être examinée plus attentivement.

## Introduction

Patients with end-stage kidney disease undergoing hemodialysis (HD) are known to have moderate to severe cognitive impairments, which may go undiagnosed unless formal testing is performed.^1^ When examined, patients demonstrate global neurocognitive impairment,^1-3^ with particular deficits in the domains of executive function,^2,4^ memory,^4^ and attention.^4^ Despite the high prevalence of neurocognitive impairment in patients undergoing HD, the underlying cause is unknown.

Although the causes of cognitive impairment are multifactorial, it is possible that the process of HD causes detrimental effects to the brain. Particularly, HD may increase the incidence of stroke,^5^ cause systemic circulatory stress by affecting vulnerable perfusion-dependent vascular beds,^6^ and produce significant shifts in acid/base balance.^7^ Decreases in white matter integrity,^8^ along with an increased incidence of silent infarcts,^9^ have also been documented. Prospective serial monitoring of cerebral perfusion has not been performed in patients initiating HD.

Regional cerebral oxygenation (rSO2) is a surrogate marker for cerebral perfusion, and may provide insight into the deleterious cerebral effects of HD. Regional cerebral oxygenation can be measured using near-infrared spectroscopy (NIRS), which has been extensively used in cardiac surgery,^10^ and has been shown to linearly correlate with computed tomography (CT) perfusion,^11,12^ Near-infrared spectroscopy was selected for this study as it is relatively inexpensive in comparison with magnetic resonance imaging, is portable and requires little training to set up, is noninvasive, and allows for continuous monitoring while producing a real-time assessment of the oxygenation status of hemoglobin.^13,14^ Studies comparing rSO2 before dialysis initiation with control subjects demonstrated that dialysis patients had lower rSO2 levels than controls, with values being 40% to 55% and 70%, respectively.^14-17^ In addition, lower rSO2 levels have been measured in the frontal lobe and extremities during HD, suggesting that tissue ischemia may be occurring.^17^ Finally, rSO2 levels have also been negatively correlated with blood pH, diabetes, and the duration of time the patient has been on HD.^18^ In addition to NIRS, studies using other imaging modalities have investigated the impact of dialysis on the brain. In particular, a study using diffuser tensor imaging was able to investigate the structural integrity of patients receiving HD and determined that cognitive deficits were correlated with white matter damage.^19^ In addition, positron emission tomography–CT determined that global cerebral blood flow decreased by 10±15% by the end of a dialysis session,^20^ and Doppler studies have confirmed this decline and further added that cerebral blood flow declines were related to intradialytic cognitive impairment.^21^

Our overall hypothesis is that repetitive cerebral hypoperfusion during HD contributes to the neurocognitive impairment reported in patients undergoing HD. However, the feasibility of prospectively monitoring rSO2 in patients initiating HD however is unknown. Therefore, the primary objective of the study was to assess the feasibility of serially monitoring rSO2 weekly for 1 year after HD initiation. Second, relationships between rSO2 with hemodynamic and dialysis parameters were examined, along with changes in patient neurocognitive performance after the initiation of dialysis.

## Methods

### Study Design, Location, Participants, and Data Acquisition

This prospective observational study was performed at an academic teaching hospital. Eligible patients were recruited from a multidisciplinary chronic kidney disease (CKD) clinic if they were >17 years old and had chosen in-center HD as their kidney replacement therapy. Patients were excluded if they had any documented history of stroke or neurodegenerative disease. Patients who also chose home-based kidney replacement therapy (home HD or peritoneal dialysis [PD]) were also excluded. All patients provided informed consent before any monitoring or neuropsychological testing was performed. The cause of CKD and comorbidities were abstracted from clinic reports. Ethnicity, primary language, handedness, and highest education were collected at the first neurocognitive assessment. All hemodynamic and dialysis parameters were collected by Cordiax 5008 dialysis machine in 15-minute intervals during the dialysis sessions. These parameters included heart rate (HR), blood pressure, total fluid removed, ultrafiltration rate, dialysate temperature, and predialysis and postdialysis weight. The study was approved by Queen’s University and Affiliated Hospitals Health Sciences Research Ethics Board.

### Feasibility Definition

Feasibility was defined as (1) successful patient enrollment (defined as >1 patient/mo), (2) successful informed consent (defined as >70%), (3) high rSO2 data capture rates (>90%), and (4) rSO2 sensor monitoring tolerability.

### Regional Cerebral Oxygenation Monitoring

The CASMED Foresight cerebral oximeter was used to capture rSO2 during HD. Regional cerebral oxygenation monitoring occurred on the first dialysis day of the week (Monday or Tuesday dialysis schedule dependant) for the patients’ first year of dialysis. The CASMED oximeter captures and displays the patient’s rSO2 level every 2 seconds for the 4 hours they receive their dialysis treatment. Each sensor is 5 cm in length and is placed directly on the forehead, allowing near-infrared light to penetrate roughly 2.5 cm into the frontal cortex.^22^

### Neurocognitive Assessment

Neurocognitive assessments were performed at 3 time points: prior to initiation of HD, 3 months, and 1 year after HD initiation. The assessment included the Repeatable Battery for the Assessment of Neuropsychological Status (RBANS), and a robotic assessment using the Kinarm Endpoint Lab.^23^ This battery has recently been proven feasible in quantifying neurocognitive impairment in those with CKD,^24^ and took approximately 1 hour and 20 minutes to complete.

The RBANS and Kinarm task components and descriptions are itemized in Table 1. The RBANS tests 5 domains: immediate memory, delayed memory, attention, visuospatial, and language, and is summarized with a composite score (total scale score). Kinarm Standard Tests were used to quantify sensory, motor, and cognitive functions associated with the upper limbs.^25^ This robotic assessment provides objective and quantitative measures of neurocognitive performance, and has been shown to detect more impairment in patients with CKD, compared with traditional testing.^24^ Kinarm Task Scores are a composite summary score characterizing patient performance by incorporating a number of spatial and temporal parameters. These Task Scores are corrected for age, sex, and handedness, that is then transformed into a positive score, where 0 denotes best performance possible and 1.96 denotes the 95th percentile for neurologically healthy performance. Repeatable Battery for the Assessment of Neuropsychological Status scores are normalized by age only. We defined impairment as scores beyond the 95th percentile, as it allows for greater certainty of true impairment. Therefore, a score of <75.25 (1.65 SD) on the RBANS and a score of >1.96 on Kinarm were considered impaired. All individual patient scores are included in Table 1 of the Supplemental Data.

**Table 1.** Neurocognitive Battery Tests With Task Components and Descriptions.

Neurocognitive test	Domain/task	Task components and description
RBANS	Attention	Digit span, symbol coding
	Immediate Memory	List learning, story memory
	Delayed memory	List recall, list recognition, story recall, figure recall
	Visuospatial	Figure copy, line orientation
	Language	Picture naming, semantic fluency
	Total scale score	A summary score based on the performance on all the RBANS domains. It represents the global performance on the RBANS assessment.
Kinarm	Arm position matching	The robot moves 1 of the patient’s arms. Patients are then asked to mirror match this position with the other arm. This task measures proprioception and somatosensory.
	Ball on bar	A bar appears between the patient’s 2 hands with a ball on top of it. The patient is then asked to move the ball into targets that appear on the screen. There are 3 levels with increasing difficulty, where the ball goes from being fixed in position to being able to freely move and fall off the bar (depending on how level the patient keeps the bar). This measures visuomotor skill, motor range, and coordination.
	Object hit	Paddles appear at the patient’s hands and they are instructed to hit as many balls (which fall from the top of the screen) away as possible. As the task progresses, the quantity and speed at which the balls fall increases. Measures attention and visuomotor capabilities.
	Object hit and avoid	Similar to OH, but this time, 2 shapes are shown to the patient which they are instructed to hit while avoiding all the other distractor targets. Again, as the task progresses, the quantity and speed at which the targets fall increase. Measures attention, visuomotor, and executive function.
	Visually guided reaching	A target appears on the screen and the patient is asked to move the dot that represents their hand into the target as quickly and accurately as possible. A measure of visuomotor capabilities.
	Reverse visually guided reaching	Similar to VGR, but the light representing their hand now moves in the opposite direction of the arm movement. Measures visuomotor capabilities and executive function.
	Spatial span	Patient is shown a sequence and is then asked to replicate it. The length of the sequence depends on whether the patient got the previous sequence correct or not. A measure of working memory.
	Trail Making Test A	The patient is asked to connect the numbers 1-25 in order as quickly as possible. The number arrangement is predetermined and no connecting lines may overlap. Measures visuomotor and attention.
	Trail Making Test B	Similar to TMT-A, but the patient is now asked to alternate between numbers and letters (1-A-2-B-3) as quickly as possible. Like TMT-A, the arrangement is predetermined and no connecting lines may overlap. A measure of visuomotor, attention, and executive function.

*Note*. The Repeatable Battery for the Assessment of Neuropsychological Status (RBANS) and Kinarm domains and tasks are assessed, with task components and descriptions. OH = object hit; VGR = visually guided reaching; TMT-A = Trail Making Test A.

### Data Analysis

We used descriptive statistics to describe neurocognitive performance. In addition, significant change in performance on Kinarm was calculated using significant change thresholds and confidence intervals, which is described in Simmatis et al.^26^ To determine whether hemodynamic and dialysis parameters were related to rSO2, Pearson correlation plots were created using *P* values with a 95% confidence level. We acknowledge that due to the vast amount of data available to analyze for each individual patient, spurious associations may be identified. Therefore, we will characterize the associations based on Cohen’s work^27^ as small association (questionable clinical significance; *r* < .2), moderate association (*r* = .3-.4), or strong association (*r* > .5). All analyses were performed using R Studio.^28^ For completeness, both the Pearson correlation coefficients (*r* value) and *P* values are included as Supplemental Data (Table 2), for both the cohort as a whole and per individual patient.

**Table 2.** Patient Characteristics.

Patient characteristic	Total cohort (N = 6)
Age (mean, range)	68.5 (51-88)
Sex (N, %)	
Male	5 (83.33)
Female	1 (16.67)
Handedness (N, %)	
Right	6 (100)
English as a second language (N, %)	
No	6 (100)
Ethnicity (N, %)	
Caucasian	6 (100)
Highest reported education (N, %)	
Grade 8-11	1 (16.67)
Grade 12	3 (50.00)
University	1 (16.67)
Masters	1 (16.67)
Cause of CKD (N, % of total N)	
Diabetic nephropathy	5 (83.33)
Hypertension	1 (16.67)
Focal segmental glomerulosclerosis	1 (16.67)
Comorbidities (N, % of total N)	
Diabetes	5 (83.33)
Hypertension	5 (83.33)
Cardiovascular^a^	4 (66.67)
Sleep apnea	3 (50)
Other^b^	8

*Note*. Summary of patient clinical and demographic variables. Cause of chronic kidney disease (CKD) was multifactorial for most patients; therefore, if the cause of CKD was determined to be both diabetes and hypertension, it was accounted for in both sections.

a Cardiovascular comorbidities: coronary heart disease (N = 3) and peripheral vascular disease (N = 1).

b Other comorbidities: dyslipidemia (N = 3), hyperthyroidism (N = 2), proteinuria (N = 1), albuminuria (N = 1), and left nephrectomy (N = 1).

## Results

### Feasibility

**Figure 1.**
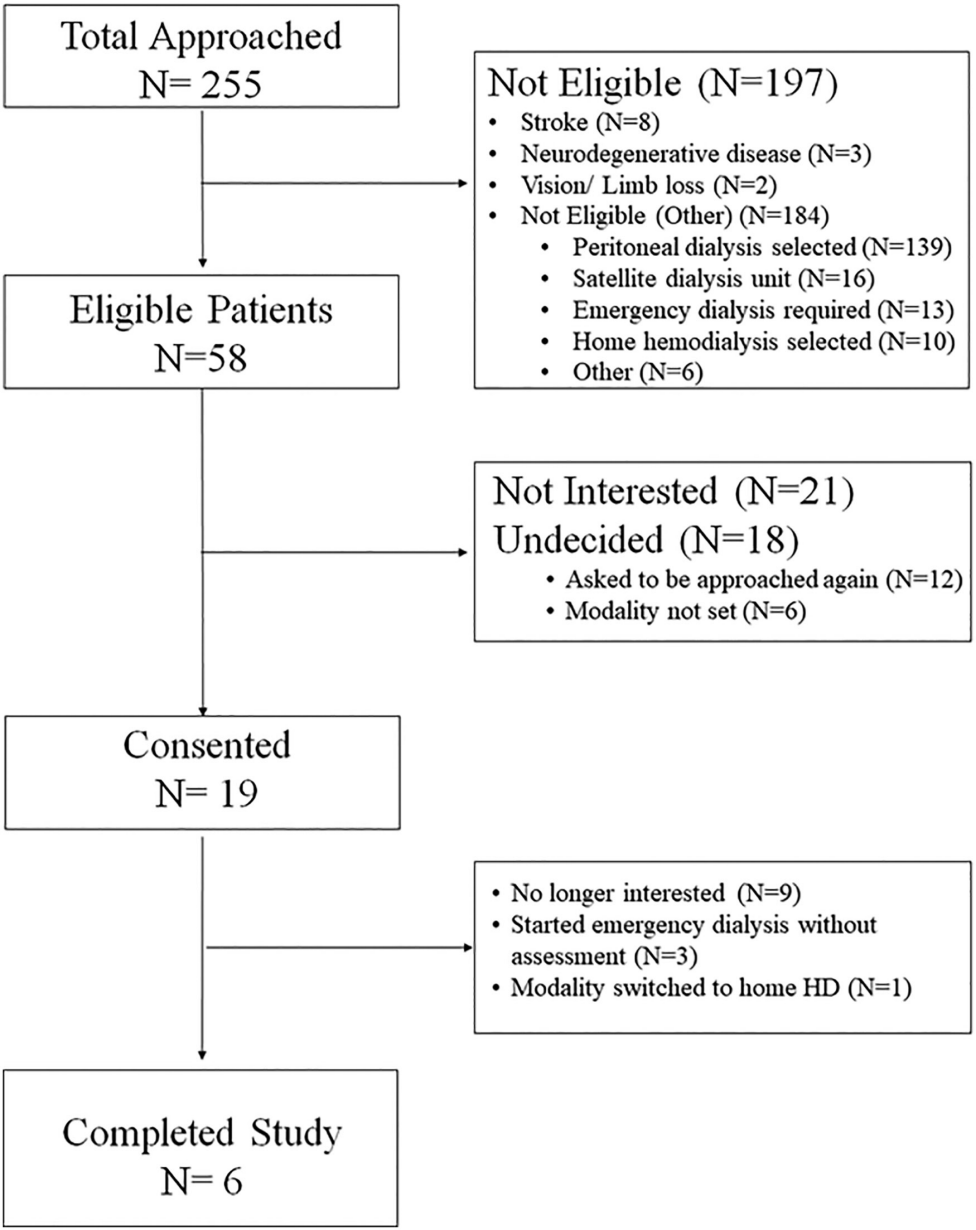
Consort diagram from June 2015 until March 2019. *Note*. HD = hemodialysis.

Participants were enrolled from June 2015 until March 2019. During that time, 255 patients were assessed, and 58 were considered eligible (Figure 1). Most patients were not eligible due to their renal replacement therapy of choice being PD (Figure 1). Of the 58 patients, 21 were not interested, 12 asked to be approached again, 6 had not decided on their dialysis modality when approached, and 19 (33%) consented (Figure 1). Of those 19, 9 withdrew from the study, 3 required unexpected emergency dialysis before completing their neuropsychological assessment, and 1 switched to home HD (Figure 1). Thus, 6 patients completed the study in its entirety (Figure 1). Therefore, roughly one third of the eligible population was enrolled (19/58), and of those enrolled, roughly one third completed the study (6/19). The overall recruitment rate was 0.40 patients/mo.

When examining the data capture rates of rSO2 between the 6 patients, there was a total of 243 rSO2 data sessions recorded. Five patients completed a year of rSO2 monitoring, while 1 had 8 months of monitoring due to the need to transfer to a satellite dialysis clinic. Of the 243 sessions, it was determined that 91.4% (222/243) were successful. A successful data capture session was defined as any session that had less than 5 minutes of rSO2 data missing, equating to approximately 2% of the data session missing. The main reason for missing data was due to the sensor lifting from the forehead causing ambient light to interfere with the rSO2 reading. Finally, during each dialysis session, the patient was monitored to ensure that there were no adverse reactions from the sensor’s adhesive. Throughout the study, no reactions were detected.

### Patient Characteristics

Patient demographics are located in Table 2. The mean age of the participants was 68.5 years, all spoke primarily English, and were right-hand dominant (Table 2). Five of the 6 patients were male and had a highest education of grade 12 and above (Table 2). Diabetic nephropathy was the primary cause of CKD (5/6), along with hypertension (1/6) and focal segmental glomerulosclerosis (1/6) (Table 2). Diabetes (5/6), hypertension (5/6), and cardiovascular disease (4/6) were all common comorbidities in the cohort (Table 2). This cohort’s baseline characteristics were comparable with regard to age, education level, history of diabetes, or hypertension when compared with our published data of CKD stage 5 patients from the same center.^24^

### All Patients Showed Consistent Positive Correlations Between rSO2 and Mean Arterial Pressure

**Figure 2.**
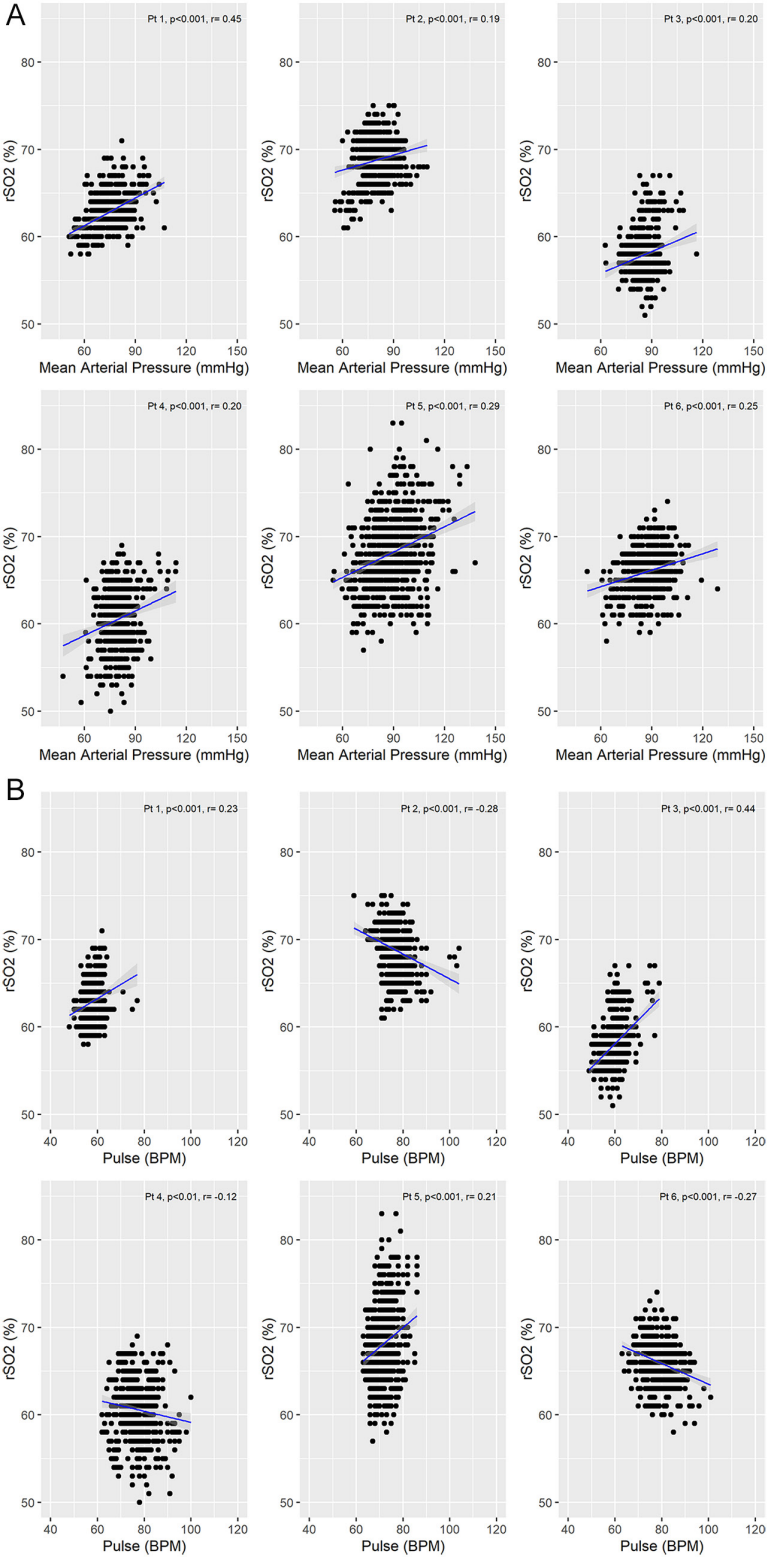
Correlations between rSO2 and hemodynamic parameters for all patients: (A) Correlations between rSO2 and MAP. MAP was calculated by one third of systolic blood pressure + two thirds of diastolic blood pressure. (B) Correlations between rSO2 and pulse. *Note.* On all graphs, the blue line represents the line of best fit, with the gray shaded area depicting the 95% confidence interval. MAP = mean arterial pressure; rSO2 = regional cerebral oxygen saturation; mm Hg = millimeter of mercury; pt = patient; BPM = beats per minute.

The relationship between the hemodynamic parameters of mean arterial pressure (MAP) and HR with rSO2 was investigated. Regional cerebral oxygenation and MAP were positively correlated for all study patients (*r* range = .19-.45) (Figure 2A). Although there was individual patient variability regarding HR (pulse) and rSO2, all 6 patients showed a significant correlations (both positive and negative, *r* range = −.28 to .44) (Figure 2B). All individual and group correlation *r* and *P* values are summarized in Table 2 in the Supplemental Data, along with a summary figure of the combined groups’ performance per individual patient (Figure 1, Supplemental Data).

### Total Fluid Removed Showed a Moderate Correlation With rSO2

**Figure 3.**
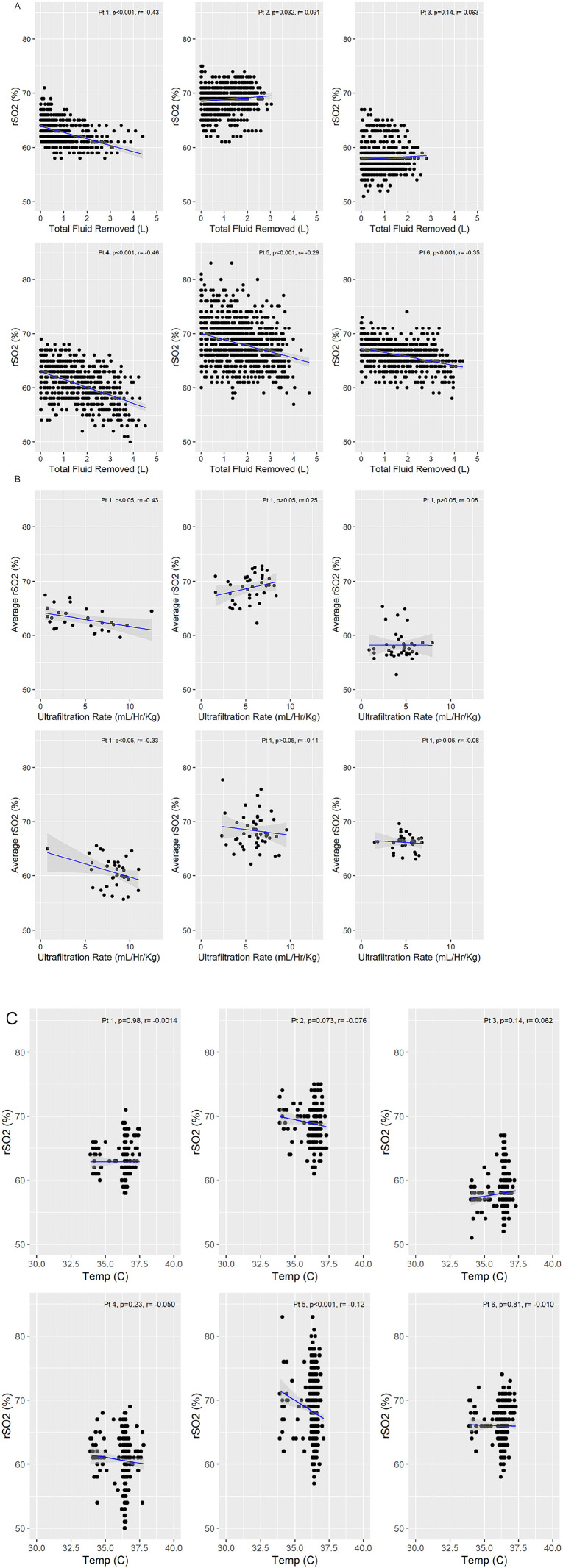
Correlations between rSO2 and dialysis parameters: (A) Relationship between rSO2 and the total fluid removed. (B) Correlations between rSO2 and the ultrafiltration rate taking into account the patient’s body weight (mL/h/kg). (C) Correlations between rSO2 and dialysate temperature. *Note.* The blue line represents the line of best fit, with the gray shaded area depicting the 95% confidence interval. rSO2 = regional cerebral oxygen saturation; pt = patient; Temp = dialysate temperature.

The dialysis parameters of total fluid removed, dialysate temperature, and ultrafiltration rate were investigated with relation to rSO2. Negative associations were found between total fluid removed and rSO2. These correlations were only present in the 4 patients who, on average, had more than 3 L of fluid removed (*r* range = −.43 to −.29) (Figure 3A). Both dialysate temperature and ultrafiltration rate showed few correlations with and rSO2 at the patient level. Regarding the ultrafiltration rate, only 2 patients exhibited moderate negative correlations (*r* = −.43, −.33) and the rest showed no association (Figure 3B). Dialysate temperature was not associated with rSO2 in 5/6 patients in our cohort (Figure 3C).

### Dialysis Vintage Was Strongly Correlated With rSO2

**Figure 4.**
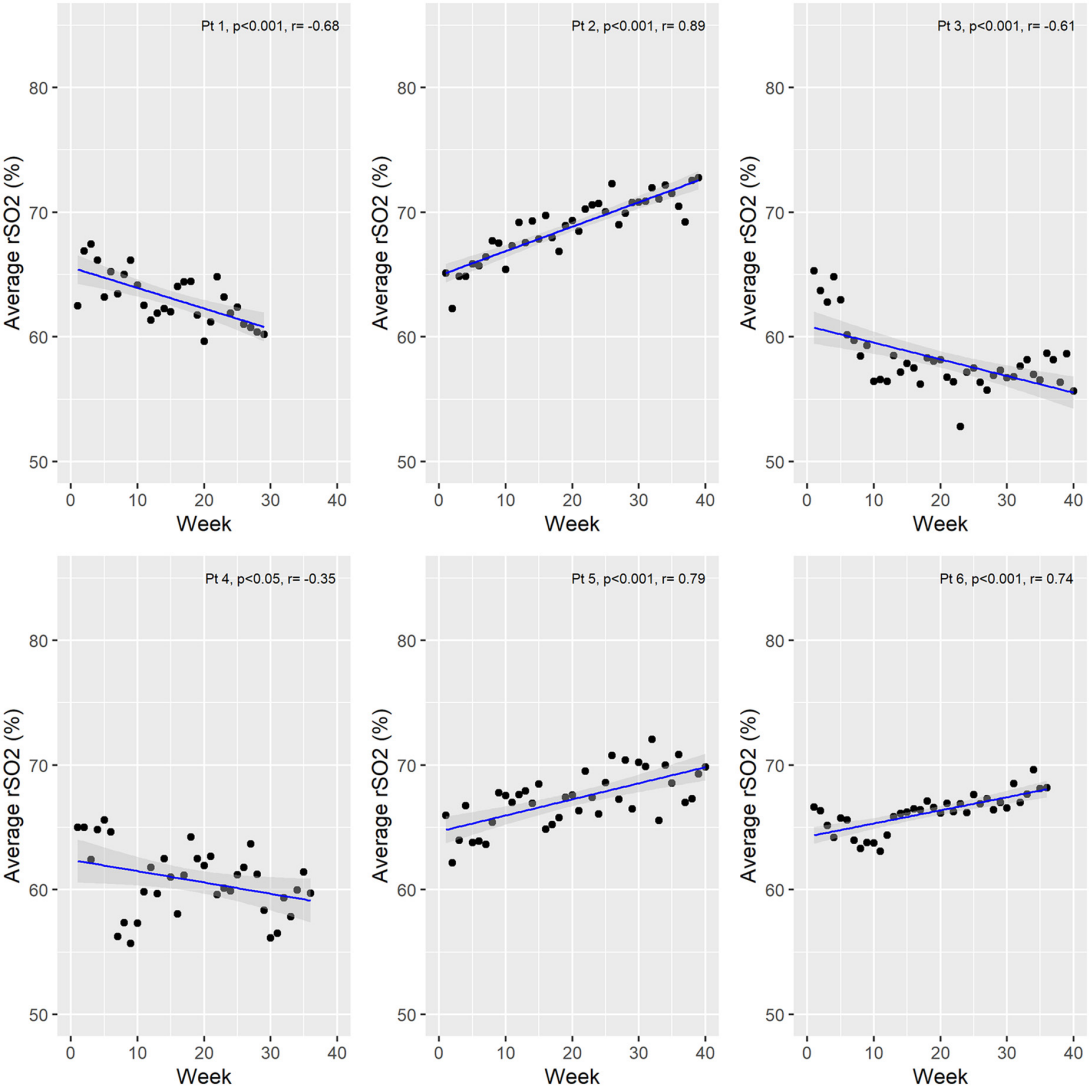
Correlations between the average weekly rSO2 and dialysis vintage. *Note.* Patient 1 only received roughly 8 months on rSo2 monitoring before switching to a satellite dialysis clinic. rSO2 = regional cerebral oxygen saturation; pt = patient.

The effects of dialysis vintage on rSO2 was also assessed. Although there was individual patient variability, all 6 patients demonstrated strong correlations (*r* range = −.68 to .89) (Figure 4).

### Neurocognitive Outcomes

**Figure 5.**
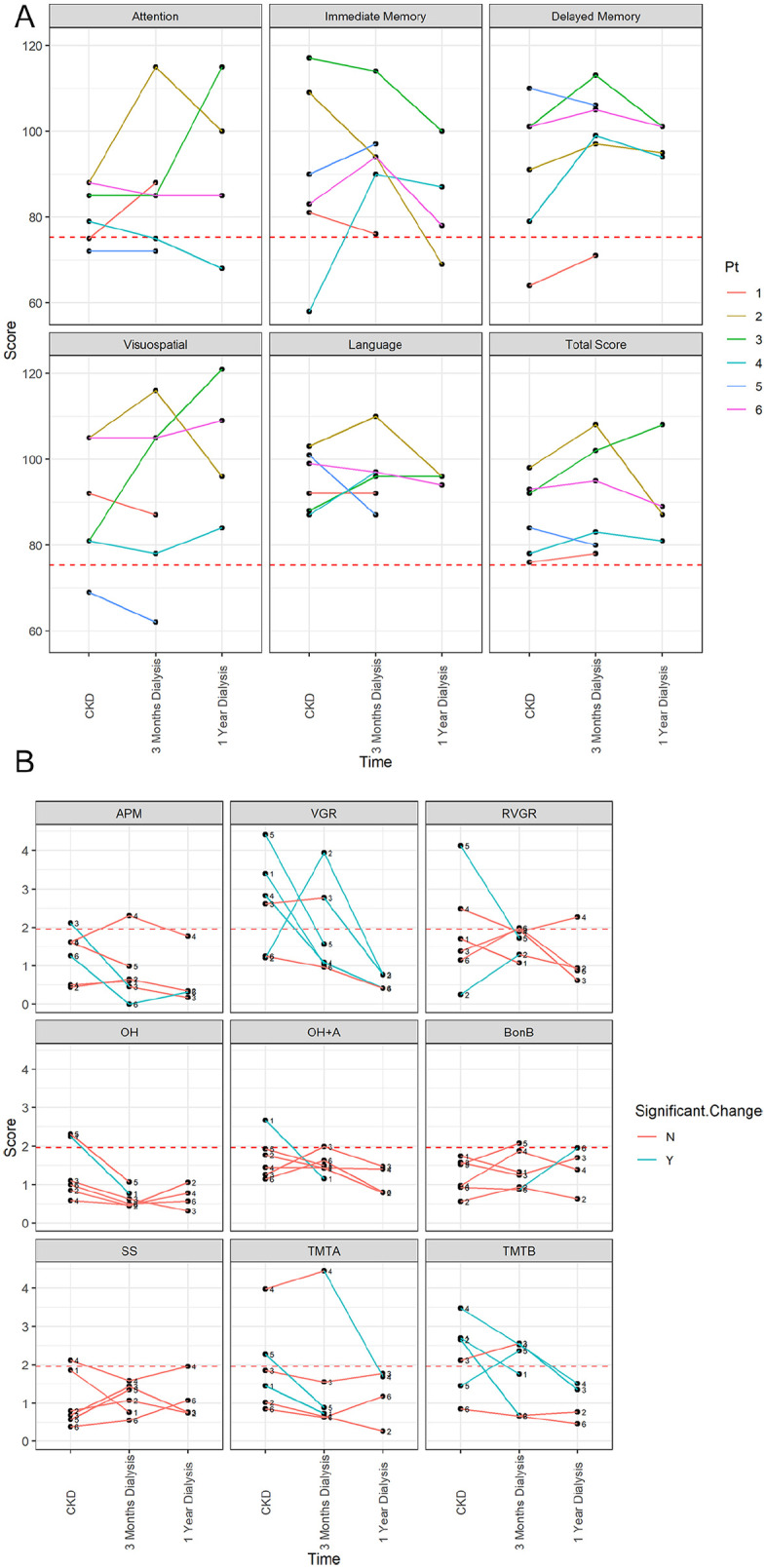
Neuropsychological assessment for all 6 patients. Each line represents a patient’s performance across the testing time points: before dialysis (stage 5 [CKD]), 3 months, and 1 year after dialysis initiation. Patients 1 and 5 did not complete their 1-year assessments as 1 patient was transferred to a satellite dialysis clinic, and the other expired before the assessment could be completed: (A) The Repeatable Battery for the Assessment of Neuropsychological Status (RBANS) Scores. The dashed red line represents the 1.65 SD cutoff. Anything below the red dotted line (a score of 75.25, the 95th percentile of healthy performance cutoff) indicates impairment. Each patient’s performance is denoted by a different color. (B) Robotic Kinarm assessment. The dashed red line represents the impairment cutoff of 1.96 (the 95th percentile of healthy performance cutoff). Anything above the dashed red line indicates impairment. Each patient’s performance is labeled by their patient number. Significant difference in performance is denoted by the blue line between test points. A red line indicated no significant change in performance. *Note.* CKD = chronic kidney disease; pt = patient; APM = arm position matching; VGR = visually guided reaching; RVGR = reverse visually guided reaching; OH = object hit; OH+A = object hit and avoid; BonB = ball on bar; SS = spatial span; TMT-A = Trail Making Test A; TMT-B = Trail Making Test B.

All patients completed the RBANS (standardized assessment) and Kinarm (robotic assessment) at the baseline (stage 5 CKD) and 3-month assessment intervals. However, 2 patients did not complete the 1-year follow-up assessment, as 1 patient transferred to a satellite clinic and the other expired. No impairment was detected for any patient at any of the time intervals for the total scale score on the RBANS, which is indicative of global performance (Figure 5A). Regarding the attention domain, 2 patients were impaired at baseline. The domains of visuospatial, immediate memory, and delayed memory demonstrated 1 patient as impaired at baseline (Figure 5A). After 3 months of dialysis, performance was almost identical with the exception of the immediate memory domain, in which no patient was impaired (Figure 5A). At the 1-year follow-up, only the domains of attention and immediate memory had 1 patient as impaired (Figure 5A).

The Kinarm assessment identified more impairment at baseline, with 8/9 tasks categorizing at least 1 patient as impaired (Figure 5B). The task of visually guided reaching which measures visuomotor capabilities showed the most impairment at baseline with 4/6 patients scoring outside the normative range (Figure 5B). Tasks measuring proprioception, visuomotor, attention, memory, and/or executive function showed 1 to 3 of the 6 patients as impaired (Figure 5B). Once dialysis was initiated, improvement was seen on all but 3 tasks (arm position matching [1/6], object hit and avoid [1/6], and Trails Making Test B, [3/6]) (Figure 5B). One task (ball on bar) which measures motor range and visuomotor capabilities was impaired at 3 months for 1 patient but not at baseline (Figure 5B). All tasks except reverse visually guided reaching (1/4) demonstrated no clinical impairment in the 4 individuals who completed the 1-year testing (Figure 5B). When investigating individual significant change in performance across the time intervals for the Kinarm, it was determined that the tasks of visually guided reaching (3/6 at 3 months, 2/6 at 1 year), and Trails Making Test A (2/6 at 3 months, 1/6 at 1 year) and B (3/6 at 3 months, 2/6 at 1 year) demonstrated the most improvement after dialysis initiation. Arm position matching also demonstrated that 2/6 patient’s performance significant improved along with 1/6 patients on object hit and object hit and avoid after 3 months of dialysis.

## Discussion

The mechanisms that contribute to the cognitive impairment in patients undergoing HD are poorly understood. This study investigated whether repetitive cerebral hypoperfusion contributed to cognitive impairment in patients initiating HD, by determining the feasibility of serially monitoring rSO2 for a patients’ first year of HD. Although our data capture rate was 91.4%, the low enrollment and consent rates at a single-center were identified as the main impediments to the feasibility of this study. Individual patient variability was present throughout the study with regard to cognitive performance, hemodynamic and dialysis parameters, and rSO2.

This study was ultimately unfeasible due to the low consent (33%) and recruitment (0.4 patients/mo) rates at a single-center level. Notably however, of 255 eligible patients, 197 were ineligible due to a previous neurological disease or selection of at home HD or PD, which drastically limited our potential study pool. Interestingly, however, poor patient enrollment has already been highlighted as a factor that limits the extent of clinical and transitional research in nephrology.^29^ A survey on medical research participation in individuals without CKD determined that although most participants reported they were willing to participate in research (59%-86%), only 11% participated.^30^

The average consent and enrollment rates for patients undergoing HD to participate in research are scarce, but would be important to identify, as it would provide benchmarks for future feasibility studies. To understand our feasibility metrics, we have identified a convenience sample of 12 feasibility studies regarding patients undergoing HD^31-42^ that contextualize our findings. Of the studies that provided study durations, the calculated enrollment rates ranged from 2.4 to 40 patients/mo.^33,34,38-42^ Consent rates ranged from 29% to 72%, with a study completion rate range from 70% to 100%.^31,33,34,36,39-42^ Therefore, our enrollment rate was lower than that of these studies at 0.4 patients/mo, but our consent (33%) rate was comparable.

Because low enrollment and consent rates were the major factors affecting the feasibility of our study, strategies to improve these metrics are important to identify. First, 10 patients were considered ineligible because they had selected home HD. Home rSO2 monitoring could be considered to account for patients who choose to dialyze at home, which could increase the enrollment rate and generalizability, as both HD and PD patients could be enrolled. Second, 3 consented participants were considered ineligible because they required emergent HD prior to completing baseline neurocognitive assessment. Determining when a patient will require dialysis is difficult due to the complexity of the disease.^43^ Therefore, a strategy to improve enrollment of these individuals could be to implement screening measures such as the Mini-Mental State Examination (MMSE) at the time of consent. However, a caveat with screening tools, such as the MMSE, is they may not be sensitive enough to detect subtle cognitive deficits.^44^ For instance, the RBANS did not detect any global impairment in our cohort, whereas most patients demonstrated objective and quantifiable impairment with the robotic testing. Another way to include patients who require emergency dialysis would be to recruit patients who have been on dialysis for less than a month. However, the challenge with this is that it has been determined that a single episode of dialysis may affect cognitive performance.^31^ Finally, challenges regarding recruitment may be in part remedied by the patient’s radius to the dialysis unit and center. Our center receives patients from a vast geographic area and includes multiple satellite units. More urban centers may have more eligible patients available to them as patients are closer to the treatment center, and these dialysis units may have a larger capacity.

Our study focused on individual data versus group data with regard to determining the associations between rSO2 and related variables. We have previously demonstrated significant interindividual variability in the association between rSO2 and the hemodynamic variables of MAP and HR in critically ill patients, which may be lost when group data are pooled.^45^ This study would suggest that patients undergoing HD have similar interindividual variability. Should cerebral hypoperfusion be identified as a risk factor for neurocognitive impairment in individuals undergoing HD, the high interindividual variability would limit any “one-size fits all” approach to optimizing cerebral perfusion during dialysis.

Surprisingly, the quantitative deficits identified with Kinarm improved after the initiation of dialysis in our cohort. This is not consistent with prior studies.^46,47^ However, the impairments that were detected (visuomotor, attention, and executive function) were similar to what we discovered in our CKD population using the same neurocognitive battery.^24^ Given the small sample size of our study, it is difficult to speculate as to why our data differs, or if this trend would remain with a larger sample. Neurocognitive improvement in this cohort may be due in part to the fact that this robotic tool is assessing neurocognitive domains not previously studied extensively in patients with CKD receiving HD. In addition, it has been postulated that the complex nature of the Kinarm tasks measures both cortical and subcortical brain areas through the use of the sensorimotor circuits which involve subcortical structures such as the basal ganglia and cerebellum, along with cortical areas such as the frontalparietal cortex known to be involved in skilled goal-directed motormovements.^48^ However, imagining would be needed to determine the precise cortical areas of activation per Kinarm task.

In addition to the small sample size, this study has several limitations. First, a major limitation of using NIRS is that the sensor only penetrates 2.5 cm^22^ through the skull into the frontal lobes. While this technology can determine the oxygenation status of cortical tissue and its immediately adjacent subcortical white matter, it likely does not assess the deeper subcortical tissues known to be affected in patients with kidney disease.^49,50^ Second, we did not screen our patients for mood disorders, such as depression, which is known to be a confounding variable for neurocognitive performance,^51^ and such information would be important to obtain in future work.

In summary, serially capturing rSO2 data during a patients’ first year of dialysis was unfeasible in a single dialysis unit, given the low consent and enrollment rates. However, data capture rates were >90% and interesting associations with respect to MAP, total fluid removed, and dialysis vintage with rSO2 were identified at the individual patient level. Future studies are needed to determine the true extent of impairments caused by HD, and how this process affects the patient’s rSO2 levels, cognition, quality of life, and mortality.

## Supplemental Material

sj-pdf-1-cjk-10.1177_20543581211010654 – Supplemental material for Cerebral Perfusion in Hemodialysis Patients: A Feasibility Study Click here for additional data file. Supplemental material, sj-pdf-1-cjk-10.1177_20543581211010654 for Cerebral Perfusion in Hemodialysis Patients: A Feasibility Study by Jessica Anne Vanderlinden, Rachel Mary Holden, Stephen Harold Scott and John Gordon Boyd in Canadian Journal of Kidney Health and Disease
