# Supplementary material for: Cerebral Perfusion in Hemodialysis Patients: A Feasibility Study
Source: Can J Kidney Health Dis. 2021 May 6;8:20543581211010654. doi: 10.1177/20543581211010654 (PMC8114747; doi:10.1177/20543581211010654)
Supplement: sj-pdf-1-cjk-10.1177_20543581211010654 – Supplemental material for Cerebral Perfusion in Hemodialysis Patients: A Feasibility Study [file sj-pdf-1-cjk-10.1177_20543581211010654.pdf]

Supplemental Data

1. Individual patient data
2. Numeric table of correlations between rSO2 and dialysis parameters
3. Figure of the entire cohorts correlations between rSO2, hemodynamic, and dialysis parameters

Individual Patient Data

		RBANS						Kinarm								
Patient	Time	Immediate Memory	Delayed Memory	Visuospatial	Attention	Language	Total Scale Score	Arm Position Matching	Ball On Bar	Object Hit	Object Hit + Avoid	Visually Guided Reaching	Reverse Visually Guided Reaching	Spatial Span	Trails Making Test A	Trails Making Test B
1	CKD	81	**64**	92	**75**	92	76	0.50	1.74	**2.25**	**2.67**	**3.40**	1.70	1.86	1.45	**2.70**
	3 Months	76	**71**	87	88	92	78	0.60	1.33	0.77	1.15	1.04	1.08	0.77	0.73	1.76
	1 Year	N/A	N/A	N/A	N/A	N/A	N/A	N/A	N/A	N/A	N/A	N/A	N/A	N/A	N/A	N/A
2	CKD	109	91	105	88	103	98	0.44	0.56	0.85	1.77	1.21	0.26	0.80	1.02	**2.65**
	3 Months	94	97	116	115	110	108	0.44	0.94	0.45	1.43	**3.94**	1.30	1.07	0.66	0.68
	1 Year	**69**	95	96	100	96	87	0.35	0.63	1.06	0.79	0.76	0.94	0.75	0.28	0.78
3	CKD	117	101	81	85	88	92	**2.12**	1.57	1.10	1.26	**2.62**	1.38	0.69	1.85	**2.12**
	3 Months	114	113	105	85	96	102	0.46	1.24	0.63	**1.99**	**2.77**	1.95	1.44	1.55	**2.56**
	1 Year	100	101	121	115	96	108	0.18	1.70	0.31	1.47	0.77	0.62	0.77	1.77	1.35
4	CKD	**58**	79	81	79	87	78	1.62	0.97	0.58	1.45	**2.83**	**2.49**	**2.12**	**3.97**	**3.47**
	3 Months	90	99	78	**75**	97	83	**2.30**	1.88	0.47	1.44	1.09	1.89	1.59	**4.45**	**2.53**
	1 Year	87	94	84	68	94	81	1.77	1.39	0.78	1.40	0.42	**2.27**	**1.96**	1.69	1.51
5	CKD	90	110	**69**	**72**	101	84	1.62	1.52	**2.32**	1.93	**4.42**	**4.12**	0.57	**2.27**	1.45
	3 Months	97	106	**62**	727	87	80	0.99	**2.07**	1.07	1.50	1.57	1.72	1.35	0.89	**2.37**
	1 Year	N/A	N/A	N/A	N/A	N/A	N/A	N/A	N/A	N/A	N/A	N/A	N/A	N/A	N/A	N/A
6	CKD	83	101	105	88	99	93	1.26	0.93	1.00	1.14	1.25	1.15	0.39	0.85	0.85
	3 Months	94	105	105	85	97	95	0.00	0.87	0.50	1.62	0.97	**1.99**	0.56	0.63	0.67
	1 Year	78	101	109	85	90	89	0.32	1.95	0.57	0.80	0.42	0.87	1.07	1.18	0.46

Table 1. Individual patient data for the neurocognitive battery. Impaired scores are in bold. Abbreviations: N/A= Patient did not complete testing at that time point.

Correlations Between rSO2 and Dialysis Parameters

Patient	Whole Cohort		1		2		3		4		5		6	
	r	p-value	r	p-value	r	p-value	r	p-value	r	p-value	r	p-value	r	p-value
Mean Arterial Pressure (mmHg)	0.16	<0.001	0.45	<0.001	0.19	<0.001	0.20	<0.001	0.20	<0.001	0.29	<0.001	0.25	<0.001
Pulse (BPM)	-0.42	<0.001	0.23	<0.001	-0.28	<0.001	0.44	<0.001	-0.12	<0.01	0.21	<0.001	-0.27	<0.001
Total Fluid Removed (L)	-0.052	<0.01	-0.43	<0.001	0.091	<0.05	0.063	NS	-0.46	<0.001	-0.29	<0.001	-0.38	<0.001
Temperature (C)	-0.023	NS	-0.0014	NS	-0.076	NS	0.062	NS	-0.050	NS	-0.12	<0.001	-0.010	NS
Ultrafiltration Rate (mL/Hr/Kg)	-0.0007	NS	-0.43	<0.05	0.25	NS	0.08	NS	-0.33	<0.05	-0.11	NS	-0.08	NS
Average rSO2/Week	0.22	<0.001	-0.68	<0.001	0.89	<0.001	-0.61	<0.001	-0.35	<0.05	0.79	<0.001	0.74	<0.001

Table 2. Cohort and individual patient correlations between regional cerebral oxygenation saturation (rSO2), hemodyanmaic, and dialysis paramerters. Abbreviations: mmHg= millimetre of mercury, BPM= beats per minute, C= celcius, L= litre, mL= millalitre, Hr= hour, Kg= kilogram, and NS= not significant.

Correlations Between rSO2 and All Parameters Investigated Combined as a Group

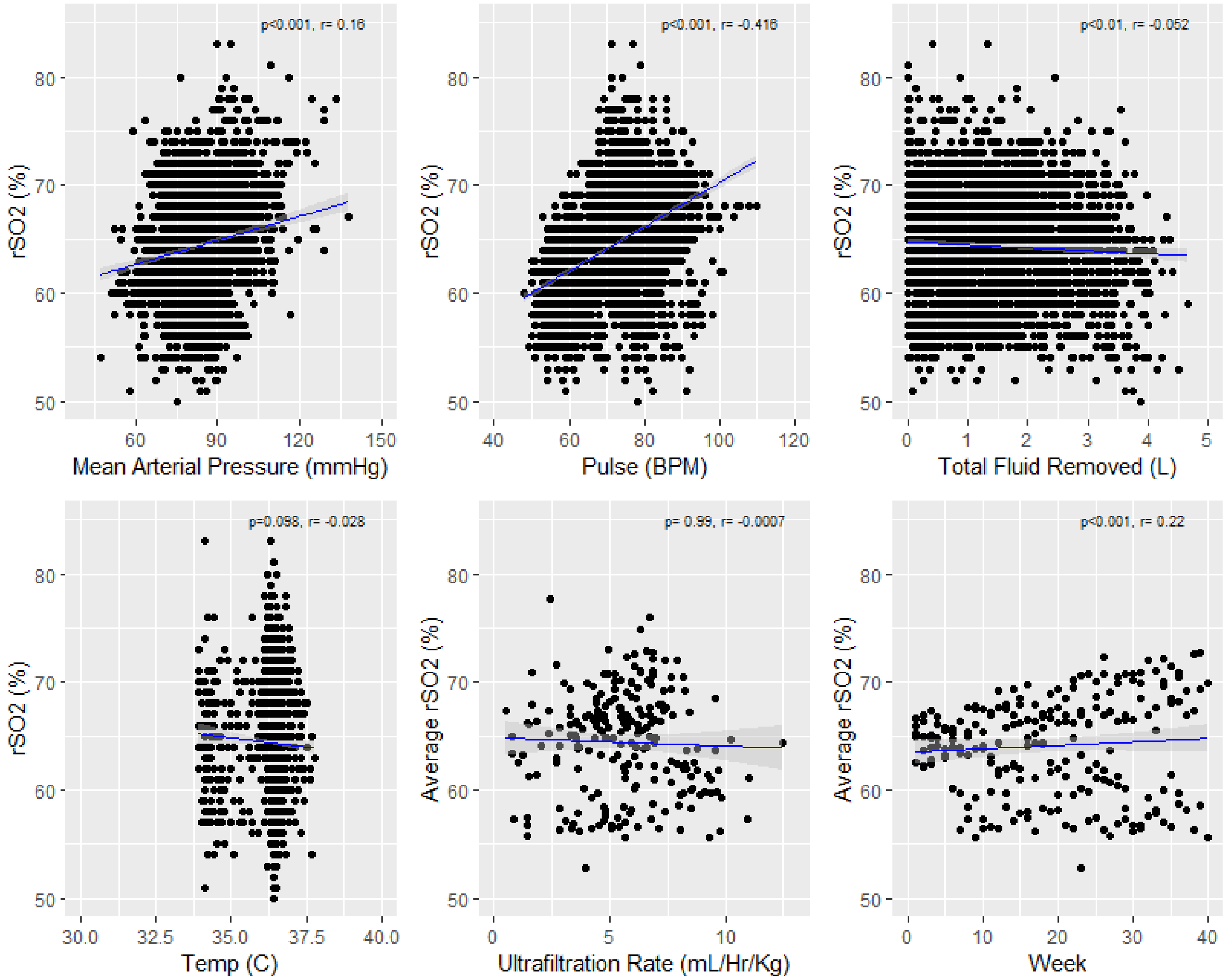

Figure 1. Correlations between rSO2, hemodynamic, and dialysis parameters of the entire cohort. Abbreviations: mmHg= millimetre of mercury, BPM= beats per minute, C= celcius, L= litre, mL= millalitre, Hr= hour, and Kg= kilogram.
